# Histone deacetylase inhibitors promote eNOS expression in vascular smooth muscle cells and suppress hypoxia‐induced cell growth

**DOI:** 10.1111/jcmm.13122

**Published:** 2017-03-07

**Authors:** Xiaoling Tan, Lan Feng, Xiaoyong Huang, Yidong Yang, Chengzhong Yang, Yuqi Gao

**Affiliations:** ^1^ Department of High Altitude Physiology & Biology College of High Altitude Medicine Third Military Medical University Chongqing China; ^2^ Southwest Eye Hospital Southwest Hospital Third Military Medical University Chongqing China; ^3^ Department of Pathophysiology & High Altitude Pathology College of High Altitude Medicine Third Military Medical University Chongqing China

**Keywords:** endothelial nitric oxide synthase, vascular smooth muscle cell, vascular remodelling, histone deacetylase inhibitor, hypoxia

## Abstract

Hypoxia stimulates excessive growth of vascular smooth muscle cells (VSMCs) contributing to vascular remodelling. Recent studies have shown that histone deacetylase inhibitors (HDIs) suppress VSMC proliferation and activate eNOS expression. However, the effects of HDI on hypoxia‐induced VSMC growth and the role of activated eNOS in VSMCs are unclear. Using an EdU incorporation assay and flow cytometry analysis, we found that the HDIs, butyrate (Bur) and suberoylanilide hydroxamic acid (SAHA) significantly suppressed the proliferation of hypoxic VSMC lines and induced apoptosis. Remarkable induction of cleaved caspase 3, p21 expression and reduction of PCNA expression were also observed. Increased eNOS expression and enhanced NO secretion by hypoxic VSMC lines were detected using Bur or SAHA treatment. Knockdown of eNOS by siRNA transfection or exposure of hypoxic VSMCs to NO scavengers weakened the effects of Bur and SAHA on the growth of hypoxic VSMCs. In animal experiments, administration of Bur to Wistar rats exposed to hypobaric hypoxia for 28 days ameliorated the thickness and collagen deposition in pulmonary artery walls. Although the mean pulmonary arterial pressure (mPAP) was not obviously decreased with Bur in hypoxic rats, right ventricle hypertrophy index (RVHI) was decreased and the oxygen partial pressure of arterial blood was elevated. Furthermore, cell viability was decreased and eNOS and cleaved caspase 3 were induced in HDI‐treated rat pulmonary arterial SMCs. These findings imply that HDIs prevent hypoxia‐induced VSMC growth, in correlation with activated eNOS expression and activity in hypoxic VSMCs.

## Introduction

Vascular remodelling is a common feature of cardiovascular diseases such as atherosclerosis and pulmonary arterial hypertension 1, 2, 3. A considerable change in the vasculature under these pathological conditions is thickening of medial layers of muscular vessels, occurring mainly due to the accumulation of vascular smooth muscle cells (VSMCs). Hypoxia, a common cause and result of cardiovascular disease, stimulates excessive growth of VSMCs and contributes significantly to vascular remodelling.

Histone deacetylase inhibitors (HDIs), which apparently inhibit histone deacetylase (HDAC) activity, suppress cell growth by modulating the expression of several genes related to the cell cycle and apoptosis 4, 5. Two HDIs, suberoylanilide hydroxamic acid (SAHA) and romidepsin (FK228), have been approved for treating cutaneous T cell lymphoma 6. Others, including butyrate, are under clinical trials. Recently, the suppressive effect of HDI on VSMC proliferation has been shown *via* the induction of p21 expression and subsequent cell cycle arrest with reduction in the phosphorylation of Rb protein at the G1–S phase 7. Either short interfering RNA‐mediated knockdown of HDAC or the pharmacological inhibition of HDAC prevented mitogen‐induced SMC proliferation 4, 8. However, the effects of HDI on hypoxia‐induced VSMC proliferation and vascular remodelling are unclear.

HDIs are a group of proteins that regulate histone acetylation in nucleosomes and mediate changes in chromatin conformation, leading to the regulation of gene expression 5, 6, 9, 10. Accumulating evidence shows that HDIs modulate histone acetylation states for the transcriptional control of proliferative genes such as p21 and p27 7, 11, 12, 13, 14. However, the epigenetic mechanism involved in the HDI‐mediated suppression of VSMC proliferation is not completely understood. Previous studies indicate that eNOS expression could be activated by the HDI, butyrate and trichostatin A (TSA) in non‐endothelial cells, including VSMCs 15, 16, 17. As previously known, nitric oxide (NO) is mainly synthesized and secreted by vascular endothelial cells *via* eNOS in physiological vasculature, which acts as an essential regulator of VSMC proliferation by inducing production of cleaved caspase 3 and p21 expression 18, 19, 20, 21, 22, 23. However, EC‐derived NO was suppressed in many pathological situations due to EC disorders and/or eNOS dysfunction 20, 24, 25. eNOS transfection or treatment with NO donors can inhibit VSMC proliferation 26, 27, 28. Furthermore, the degree of NO donor inhibition was significantly enhanced in the presence of hypoxia 28. Therefore, it is interesting to test whether HDI activates eNOS expression in hypoxic VSMCs and contributes to cell growth regulation.

In this study, we tested the effect of Bur and SAHA on eNOS gene expression in hypoxic VSMCs and determined whether eNOS gene activation in VSMCs was sufficient to suppress hypoxia‐induced VSMC proliferation. We observed that HDI treatment stimulated eNOS expression and NO secretion by hypoxic VSMCs. Their antiproliferative and pro‐apoptotic effects were attenuated by NO scavengers and siRNA‐mediated eNOS knockdown. Furthermore, induction of p21 expression and cleaved caspase 3 by HDI in hypoxic VSMCs was decreased by NO scavengers and siRNA‐mediated eNOS knockdown. Finally, we observed that Bur prevented the thickening and collagen deposition in the pulmonary artery (PA) wall in a rat model of hypobaric hypoxia‐induced vascular remodelling (simulating high altitude at 5000 m) and protected the function of the cardiovascular system with the elevation of PaO_2_ and the decreased right ventricle hypertrophy index (RVHI). Cell viability was decreased and the expression of eNOS and cleaved caspase 3 was induced in HDI‐treated rat pulmonary arterial SMCs (rPASMCs).

## Material and methods

### Cell culture and experimental treatment

The A10 SMC line was purchased from ATCC and cultured in DMEM/F12 (Hyclone) containing 10% foetal bovine serum (Gibco) and 100 μg/ml Pen/Strep (Gibco) at 37°C with 5% CO_2_ and 95% air.

Isolation and culture of pulmonary arterial smooth muscle cells (PASMCs) was performed as previously described 29. Eight male Wistar rats were used for each independent isolation. All protocols and surgical procedures were approved by the Institutional Animal Use Committee of the Third Military Medical University and were in accordance with the guidelines of the National Institutes of Health and the American Physiological Society. Briefly, rats were heparinized, anaesthetized (intraperitoneal injection of sodium pentobarbital, at 50 mg/kg) and killed by exsanguination. The thorax was immediately opened and the lungs were removed under sterile conditions. The intrapulmonary arteries, third to fourth generation, were dissected free of parenchyma and kept in ice‐cold Hanks’ buffer. Vascular segments were free from adventitia and were dissected open. The endothelium was then removed by gently scraping the luminal surface of the vessel under a dissecting microscope. After recovery for 30 min in cold (4°C) physiological salt solution (PSS) that contained 130 mM NaCl, 5 mM KCl, 1.2 mM MgCl_2_, 10 mM HEPES and 10 mM glucose followed by 20 min in reduced‐Ca^2+^ PSS (20 μM CaCl_2_) at room temperature, the tissues were digested at 37°C for 20 min in reduced‐Ca^2+^ PSS containing collagenase (type I, 1750 U/ml), papain (9.5 U/ml), bovine serum albumin (2 mg/ml) and dithiothreitol (1 mM). Cells were then collected and grown in DMEM supplemented with 10% FBS. Contaminating fibroblasts were separated from the PASMCs by exploiting their differential adhesive ability. After three passages at a ratio of 1:2 with trypsin, the purity of PASMCs was confirmed by the typical ‘hill and valley’ morphology and by α‐SMA immunofluorescence staining. Cells that had been passaged four to eight times were used in the following experiments. Each experiment was performed with the same batch of cells in triplicate, and three batches of cells from independent isolations were used in this study.

For the experiments, serum‐starved cell cultures were treated with Bur or SAHA in DMEM/F12 at the indicated concentration and then placed in the hypoxic chamber with 1% O_2_, 5% CO_2_ and 94% N_2_ for the indicated times. Cells incubated in the chamber with 5% CO_2_ and 95% air for the same duration were used as the normoxic controls.

### DNA content and/or cell cycle analysis

The changes in DNA content associated with cell cycle progression were quantified by propidium iodide (PI) staining 30. In brief, following the indicated treatment, cells were collected and washed twice with ice‐cold PBS and then fixed overnight in 70% ethanol at 4°C. After washing twice with cold PBS, cells were treated with DNA staining buffer (PBS containing 1 mg/ml PI and 10 mg/ml RNase A) (BD Biosciences) and incubated at 37°C in the dark for 30 min. The cells were washed and transferred to flow cytometry tubes for cell cycle analysis with a flow cytometer. The percentage of cells at G0, S or G2 phase was analysed. The percentage of cells at S and G2 phases served as an index of cell proliferation activity 31.

### EdU incorporation assay

To confirm the effects of HDI on the rate of DNA synthesis, cells were seeded at a density of 2 × 10^4^ cells per well in Corning 24‐well plates and cultured overnight. The culture medium was then removed, and cells were incubated in culture medium with 20 μM EdU, followed by the indicated treatment. Following each exposure, the cells were washed twice with phosphate‐buffered saline (PBS) and fixed in ice‐cold 4% (wt/vol) buffered paraformaldehyde for 20 min. After permeabilization using 0.5% Triton X‐100, EdU was conjugated with Apollo 643‐azide using the Cell‐Light™ EdU *In Vitro* Imaging kit (Ribobio, C10310‐2; Beijing, China) as per the manufacturer's instructions. Nuclei were visualized by Hoechst 33342 staining. EdU‐positive cells were imaged under a fluorescence microscope.

### Apoptosis analysis (annexin V–PI staining)

Apoptosis was analysed by the annexin V–PI staining assay 30. The Annexin V‐fluorescence (BD; NJ, USA) staining kit was used to detect the externalization of phosphatidylserine (an early event in apoptosis), and PI uptake was used as a marker of cell death. In brief, after treatment for the desired time, cells were collected and washed twice with ice‐cold PBS. The cells were then resuspended to achieve a density of 1 × 10^6^ cells/ml. To these, 100 μl of binding buffer containing 2.5 μl of annexin V–FITC and 1 μl PI (100 μg/ml) was added and incubated for 30 min in the dark. Finally, the samples were washed twice with ice‐cold PBS and transferred to flow cytometry tubes for analysis of apoptosis with a flow cytometer. The number of cells that were positive for annexin V, PI or both was then calculated.

### Cell viability assay

Cell viability was measured by the MTT assay. Briefly, PASMCs were plated in 96‐well plates at a density of 5000 cells per well. Serum‐starved PASMCs were treated with hypoxia or hypoxia along with 4 mM Bur. At the end of the desired duration, 10 μl MTT (0.5 mg/ml; Sigma‐Aldrich; St. Louis, MO, USA) was added and incubated for 4 hr until purple precipitate was visible. To this, 200 μl DMSO (Sigma‐Aldrich) was added and kept at room temperature in the dark for 10 min. Absorbance was then recorded at 570 nm.

### NO measurement

Following the indicated treatment, cell culture supernatants were collected and NO levels were measured within 30 min using the Griess method as recommended by the manufacturer (Beyotime; Shanghai,China). The lysates were extracted using RIPA buffer (Beyotime) supplemented with a cocktail of protease inhibitors (Roche; Basel, Switzerland). The protein concentration of the lysates was measured with a Bradford protein assay (Bio‐Rad; Hercules, CA, USA). NO levels were normalized to the protein contents of each dish and calculated as the ratio of the sum of nitrite and nitrate concentration (nmol) to the relevant protein amount (μg) in the cell cultures.

### Western blotting

Following the indicated treatment, cells were washed twice with ice‐cold PBS and cell lysates were extracted using urea lysis buffer (8 M urea, 0.001 M Tris buffer, pH 6.8, 1% SDS, 5 mM DTT, 1% Triton X‐100, 10% glycerol) supplemented with a protease inhibitor (Roche). The protein concentrations of lysates were measured using a Bradford protein assay (Bio‐Rad). Equal amounts of protein from each sample were separated by 10% SDS‐PAGE and then transferred to a PVDF membrane. After blocking with 5% milk for 1 hr, the membrane was incubated with primary antibody in blocking buffer overnight at 4°C. Following three washes with blocking buffer, the appropriate alkaline phosphatase‐conjugated secondary antibody (Jackson ImmunoResearch Laboratories) at a dilution of 1:2000 in blocking buffer was added and the membrane was then incubated for 1 h at room temperature. Antibody binding was visualized with an ECL detection kit (Thermo Fisher Scientific; MA, USA), and images were captured with a scanner using Quality One software (Bio‐Rad).

The primary antibodies and dilutions used in this study are summarized as follows: anti‐eNOS antibody (1:500, Santa Cruz Biotechnology, SC‐654; Santa Cruz, CA, USA), anti‐iNOS antibody (1:500, Santa Cruz Biotechnology, SC‐7271), anti‐β‐actin antibody (1:5000, Sigma‐Aldrich, A5316), anti‐Ac‐H4K12/8/5/1 antibody (1:500, Santa Cruz Biotechnology, SC‐34263), anti‐Ac‐H3K9/14 antibody (1:500, Santa Cruz Biotechnology, SC‐8655), anti‐caspase 3 antibody (1:500, Abcam (Cambridge, UK), ab4051), anti‐caspase 3 (cleaved) antibody (1:500, Abcam, ab2302), anti‐PCNA antibody (1:500, Santa Cruz Biotechnology, SC‐34263) and anti‐p21 antibody (1:500, Abcam, ab109520).

### Total RNA isolation and RT‐PCR

Total RNA was extracted using TRIzol reagent (Invitrogen) as per the manufacturer's instructions. RNA (1 μg) was reverse‐transcribed to cDNA using the Superscript III Third‐Strand Synthesis Kit (Invitrogen) in a reaction volume of 10 μl. Subsequently, 1 μl of cDNA product was used for amplification with the PCR amplification kit (Takara). The sequences of the specific primers for eNOS and β‐actin are listed in Table 1. The reaction was performed at 94°C for 30 sec., 56°C for 30 sec. and 72°C for 30 sec. for 36 cycles. β‐Actin served as the loading control. The amplified DNA was examined by electrophoresis and analysed using Quality One software. Relative mRNA levels were calculated as the density ratio of the target gene to β‐actin.

**Table 1:** Sequence of specific primers

Target	Primer sequence	Product size (bp)
eNOS cDNA	5′‐TGGCAGCCCTAAGACCTATG‐3′; 5′‐AGTCCGAAAATGTCCTCGTG‐3′	243
β‐Actin cDNA	5′‐TCATGAAGTGTGACGTTGACATCCGT‐3′; 5′‐CCTAGAAGCATTTGCGGTGCAGGATG‐3′	285
eNOS promoter	5′‐GCTTCCTGCTCCTTTGTGTC‐3′; 5′‐TCACCCTTGCTCCTCTCCTA‐3′	164
GAPDH promoter	5′‐TACTAGCGGTTTTACGGGCG‐3′; 5′‐TCGAACAGGAGGAGCAGAGAGCGA‐3′	150

### Knockdown with siRNA

To knock down eNOS expression, transfection was performed with siRNA targeted to eNOS. In brief, to formulate the lipid–siRNA complex, siRNA (Origene Technologies) and Lipofectamine 2000 (Invitrogen) were diluted separately in Opti‐MEM (Invitrogen). After incubation for 5 min., siRNA and Lipofectamine 2000 solutions were combined and then incubated for an additional 20 min. The siRNA/Lipofectamine 2000 complex was gently layered on to the cell cultures. After incubation for 4 hr, the cell medium was replaced with complete culture medium. After transfection for 16–24 hr, the cells were treated as indicated followed by total protein extraction. Western blotting was performed to evaluate the eNOS protein level as mentioned above.

### Chromatin immunoprecipitation (ChIP) assay

Following the indicated treatments, the protein–DNA complexes in the cells were cross‐linked using 1% formaldehyde solution in PBS on a rocking or shaking device for 15 min. at room temperature. After adding 125 mM glycine to quench the formaldehyde and rocking for 5 min. at room temperature, the cells were collected and treated with lysis buffer in a volume of 500 μl per 5 × 10^6^ cells on ice for 10 min. Sonication was performed to shear the chromatin to an average length of about 1 kb. Following refrigerated ultracentrifugation at 12,000× *g* for 10 min, the supernatants were collected and immunoprecipitation was performed using the ChIP Assay kit (Beyotime, P2078) as recommended by the manufacturer. The chromatin mixture was immunoprecipitated using anti‐Ac‐H4‐K12/8/5/1 (Santa Cruz Biotechnology, SC‐34263) antibody at 3 μg/ml, or an irrelevant antibody (Rabbit IgG, Beyotime, A‐7016). After DNA purification, the presence of the selected DNA sequence was assessed by PCR. The primers for the sequences of the eNOS promoter and GAPDH promoter are described in Table 1. GAPDH was set as the positive control and the sample loading control.

### Experimental animals and hypobaric hypoxia exposure

Male adult Wistar rats, weighing 180–200 g, were provided by the Animal Center of the Third Military Medical University and divided randomly into four groups: hypoxia with saline (*n* = 13), hypoxia with Bur (*n* = 13), normoxia with saline (*n* = 13) and normoxia with Bur (*n* = 13). Hypoxic rats were exposed to a simulated high altitude of 5000 m in a hypobaric chamber for 28 days. Others served as the normoxia control and were inbred outside the chamber (the height of Chongqing above the sea level is 308 m) and were treated either with saline or with Bur by intragastric injection. Bur was administered at a dose of 500 mg kg^−1^ day^−1^. All the rats had free access to food and water and were subjected to a 12‐hr light–dark cycle. Hypoxic rats were kept free from hypobaric treatment for half an hour each day for cleaning and intragastric injection. All the animal study protocols were approved by the Institutional Animal Use Committee of the Third Military Medical University and were in accordance with the guidelines of the National Institutes of Health and the American Physiological Society.

### Measurement of mean pulmonary arterial pressure (mPAP)

Following hypobaric hypoxia treatment, the rats were heparinized and anaesthetized (sodium pentobarbital, 50 mg/kg, intraperitoneal injection). The systolic pressure and diastolic pressure of the pulmonary artery were measured using a physiological recorder (PowerLab System, ADInstruments, Castle Hill, NSW, Australia), and mPAP was calculated. After the measurement, arterial blood was collected for PaO_2_ detection. The anaesthetized animals were killed by exsanguination, and the heart was harvested for subsequent experiments. All the procedures mentioned above were performed in a hypobaric chamber simulated as a 4000‐m plateau.

### Oxygen partial pressure of arterial blood (PaO_2_) measurement

Following the measurement of mPAP, arterial blood was drawn from the heart and used for PaO_2_ detection by a blood‐gas analyser following the manufacturer's instructions.

### Histology assay

Six hypoxic rats (three administered with saline and three with Bur) and three normoxic rats with saline were killed by perfusion with ice‐cold saline until they were clear of blood. After perfusion with 4% buffered paraformaldehyde for 30 min., the lungs and hearts were dissected and fixed in 4% buffered paraformaldehyde until the organs sank to the bottom. The areas of interest in the lungs were selected and embedded in paraffin, and 5‐μm‐thick slices were sectioned and mounted on glass carrier slides. The sections were stained with H&E to detect pathological changes or with Sirius Red solution (saturated picric acid containing 0.1% Direct Red 80 and 0.1% Fast Green FCF) to visualize collagen deposition.

### Evaluation of right ventricle hypertrophy

The right and left ventricles with the septum were isolated and were weighed individually. The right ventricle hypertrophy index (RVHI, the ratio of the right ventricle weight to the left ventricle plus septum weight) was calculated.

### Statistical analysis

The data are expressed as mean ± standard deviation (SD) values. Comparisons between two groups were made with two‐tailed *t*‐tests. Statistical analysis was performed using SPSS 10.0 for Windows. *P* < 0.05 was considered as statistically significant.

## Results

### HDI suppresses hypoxia‐stimulated VSMC proliferation and promotes apoptosis

**Figure 1:**
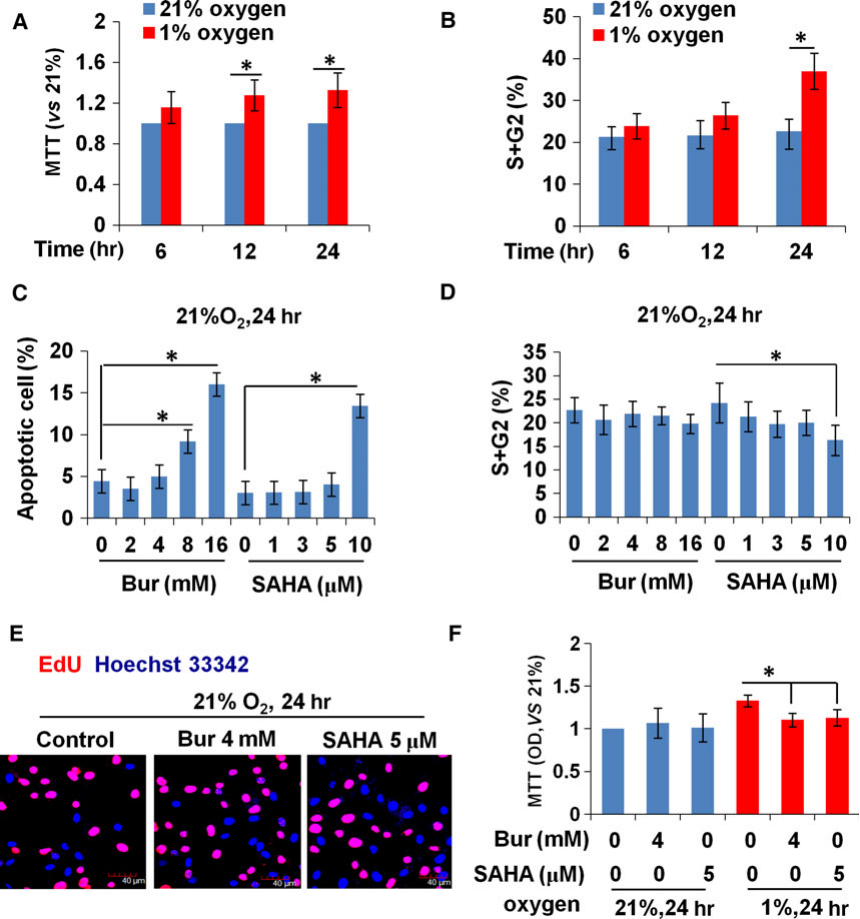
HDI at high concentrations inhibits the growth of VSMCs under normoxic conditions. (**A**) The proliferative activity of VSMCs, as detected by MTT assay, was increased significantly by hypoxia treatment for 12 and 24 hr. (**B**) Cell cycle analysis showed that the percentage of cells in the S + G2 phase was increased by 24‐hr hypoxic treatment. (**C**) The increased percentage of apoptotic cells was induced by more than 8 mM Bur or at 10 μM SAHA. (**D**) The percentage of cells in the S + G2 phase decreased in 10 μM SAHA‐treated normoxic VSMCs, but Bur had no obvious effect. (**E**) EdU incorporation was unchanged in normoxic VSMCs treated with 4 mM Bur or 5 μM SAHA. Scale bar: 40 μm. Red indicates the EdU‐positive signal and blue indicates the Hoechst 33342 staining. The experiment was performed in triplicate, and the representative images are shown. (**F**) MTT assay showed that 4 mM Bur or 5 μM SAHA suppressed the activity of hypoxic VSMCs, but had no effect on that of normoxic VSMCs.

The HDIs, Bur and SAHA, are reported to suppress PDGF‐induced VSMC proliferation 32, 33, 34. Their effect on hypoxia‐induced VSMC growth was unknown. Considering the cytotoxicity of HDI, we first tested the effects of HDI on cell growth and viability under normoxic conditions. As shown in Figure 1A and B, cell viability and cell proliferation were remarkably increased in the groups under hypoxia treatment for 24 hr. We then treated normoxia‐treated VSMCs with a series of Bur or SAHA concentrations for 24 hr. Cell cycle analysis, apoptosis by annexin V–PI staining, and MTT assay were performed. As shown in Figure 1C and D, with a Bur treatment of more than 8 mM or with SAHA treatment of more than 5 μM, apoptosis was promoted and cell cycle progress was inhibited. There were no significant changes when treated with less than 4 mM Bur or less than 5 μM SAHA, respectively, in normoxic VSMCs (Fig. 1C and D). Further studies showed that the EdU‐positive cell number in normoxic VSMCs showed no changes when treated with 4 mM Bur or 5 μM SAHA for 24 hr (Fig. 1E). However, viability of hypoxic cells was decreased by 4 mM Bur or 5 μM SAHA (Fig. 1E). Therefore, 4 mM Bur or 5 μM SAHA was used in the following experiments.

**Figure 2:**
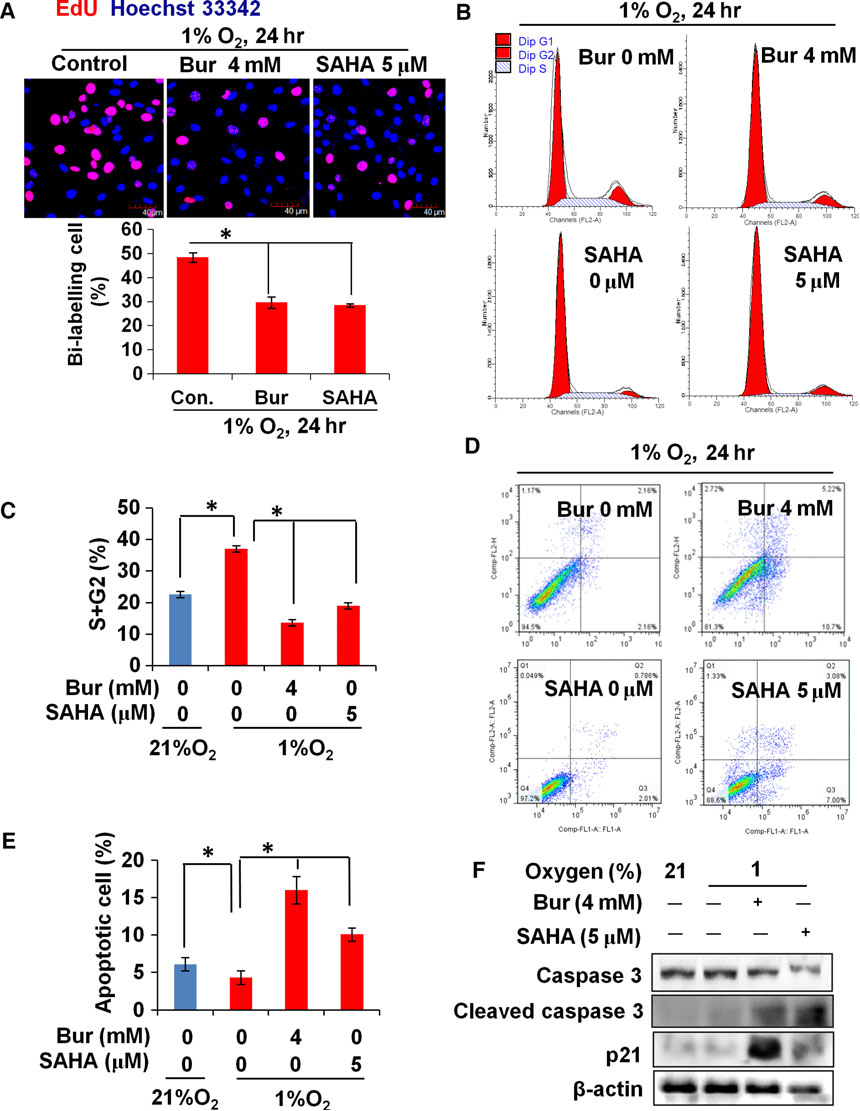
HDI suppresses proliferation and promotes apoptosis of hypoxic VSMCs. (**A**) EdU incorporation of hypoxic VSMCs was reduced significantly by the HDIs, Bur and SAHA. Scale bar: 40 μm. The experiment was performed in triplicate, and the representative images are shown. (**B**,** C**) The percentage of cells at the S and G2 phases was increased remarkably in Bur‐ or SAHA‐treated hypoxic VSMCs (*P < 0.05). The experiment was performed in triplicate, and the representative figure is shown. (**D**,** E**) The percentage of apoptotic VSMCs (indicated by positive annexin V staining and annexin V + PI double staining) was increased remarkably in Bur‐ or SAHA‐treated hypoxic VSMCs (*P < 0.05). (**F**) The protein level of cell growth regulators was detected by Western blot assay. β‐Actin served as the sample loading control. The experiment was performed in triplicate, and the representative images are shown.

We treated hypoxic VSMCs with 4 mM Bur or 5 μM SAHA and performed an EdU incorporation assay and cell cycle analysis to evaluate cell proliferation ability. The results in Figure 2A demonstrate that HDI remarkably decreased the EdU‐positive cell number in hypoxic VSMCs. The data from cell cycle analysis revealed that the percentage of cells at the S and G2 phases was significantly increased by hypoxia, but was suppressed by Bur and SAHA treatment (Fig. 2B and C). p21 is a potent cyclin‐dependent kinase inhibitor. Up‐regulation of p21 protein is a biomarker that reflects cell cycle arrest. The Western blot assay showed that HDI induced p21 expression under hypoxia (Fig. 2F). These data suggested that the proliferation ability of hypoxic VSMCs was significantly reduced when treated with HDI. To determine the effect of Bur and SAHA on apoptosis, we determined the rate of apoptotic cells by annexin V–PI staining. The data showed that 4 mM Bur or 5 μM SAHA treatment increased the rate of VSMCs that were positive for annexin V and/or PI staining (Fig. 2D and E). The levels of caspase 3 and cleaved caspase 3 are biomarkers for apoptosis activation. As shown in Figure 2F, cleaved caspase 3 was increased by HDIs in hypoxic VSMCs.

Taken together, these results indicate that Bur and SAHA suppress VSMC proliferation and promote VSMC apoptosis under normoxic or hypoxic conditions. However, hypoxic cells are much sensitive to HDI treatment and are inhibited at lower concentrations of HDIs, in which the basal growth of normoxic VSMCs is not influenced. In our study, 4 mM Bur and 5 μM SAHA were used for the following experiments.

### HDIs enhance eNOS expression and NO production in hypoxic VSMCs

**Figure 3:**
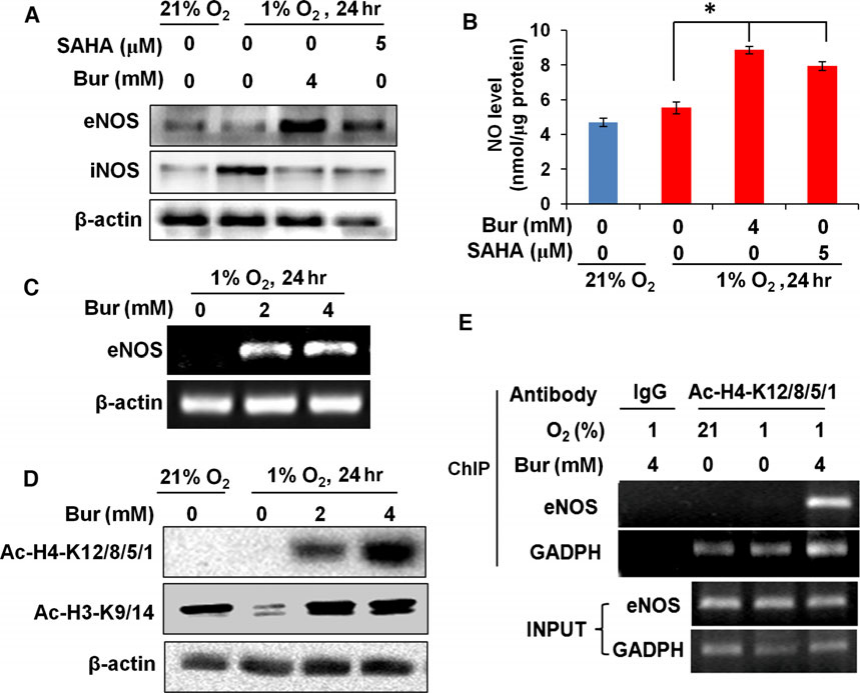
HDI enhances eNOS expression and NO secretion by hypoxic VSMCs. (**A**) Western blots showing that Bur and SAHA enhanced the protein levels of eNOS in hypoxic VSMCs. However, hypoxia‐induced iNOS expression was suppressed by HDI. β‐Actin was used as the sample loading control. The experiment was performed in triplicate, and the representative images are shown. (**B**) NO levels in the supernatant of hypoxic VSMCs were increased by Bur and SAHA treatment (*P < 0.05, *n* = 9). (**C**) Bur enhanced the eNOS mRNA levels in hypoxic VSMCs. β‐Actin mRNA levels were determined for internal normalization. The experiment was performed in triplicate, and the representative data are shown. (**D**) Total acetylation of histone H4‐K12/8/5/1 in VSMCs was barely detectable under either normoxic or hypoxic conditions, but was dramatically induced by Bur. Total acetylation of histone H3‐K9/14 was detected in VSMCs cultured under normoxic conditions, which was remarkably decreased after 24‐hr exposure to hypoxia. The addition of Bur increased the total acetylation of histone H3‐K9/14. β‐Actin was also used as the sample loading control. The experiment was performed in triplicate, and the representative data are shown. (**E**). ChIP assay was performed with anti‐Ac‐H4‐K8/12/5/1 antibody. A DNA fragment specific to the eNOS promoter was only detected in hypoxic VSMCs treated with 4 mM Bur. A DNA fragment of the GAPDH promoter was amplified as the positive control. The experiment was performed in triplicate, and the representative data are shown.

To determine the effect of HDIs on eNOS expression, we cultured VSMCs in 1% oxygen with 4 mM Bur or 5 μM SAHA for 24 hr and examined eNOS levels by Western blotting. We found that eNOS protein was hardly detected in VSMCs cultured in either 21% or 1% oxygen, consistent with previous reports. In hypoxic VSMCs, the addition of Bur or SAHA obviously increased eNOS expression (Fig. 3A). To determine whether the HDI‐enhanced eNOS expression resulted in increased NO secretion by hypoxic VSMCs, we measured the NO concentrations in cell culture supernatants by the Griess method. Upon treatment with 4 mM Bur or 5 μM SAHA, the NO levels in the hypoxic VSMC culture media were increased significantly (Fig. 3B). Under physiological conditions, eNOS is not expressed in VSMCs. However, under pathological conditions such as hypoxia, inducible NOS (iNOS) is activated and acts as the main NO producer in VSMCs. We found that hypoxia‐induced iNOS expression was suppressed by Bur and SAHA treatment, while eNOS expression was promoted (Fig. 3A). This suggests that activated eNOS expression contributes to enhancement of NO secretion by HDI‐treated hypoxic VSMCs. In summary, HDI promotes eNOS expression and NO secretion in hypoxic VSMCs.

Concerning the promoter of active genes, one common feature is acetylation at lysine residues of histones H3 and H4, which leads to a less compact and transcriptionally active chromatin. HDAC removes the acetyl groups from lysine residues, leading to the formation of a condensed and transcriptionally silenced chromatin. HDAC inhibitors block this action and thereby affect gene expression. HDIs modulate the histone modification pattern and generate a novel genomic expression profile in VSMCs 10, 11, 12, 13, 33, 50, 51, 52, 53. To further confirm the induction of eNOS by HDIs and explore its mechanism, we examined the acetylation of histones H3 and H4 on the eNOS promoter region under Bur treatment. We first confirmed that eNOS mRNA expression was enhanced by Bur (Fig. 3C), further indicating that Bur induced transcriptional activation of eNOS in hypoxic VSMCs. Histone H4 acetylation at lysine 12 (K12) and histone H3 acetylation at lysine 9 (K9) are required for the specific transcription of eNOS in endothelial cells 15, 16, and HDI induces eNOS expression in non‐EC cells 16. To explore the effects of Bur on histone H4‐K12 and H3‐K9 acetylation in VSMCs, we treated hypoxic VSMCs with 2 or 4 mM Bur for 24 h, followed by examination of acetylation levels using respective specific antibodies. Acetylation of H4 at K12/8/5/1 was barely detectable and was not affected by hypoxia (Fig. 3D). Importantly, the addition of Bur remarkably increased the acetylation levels of H4 at K12/8/5/1 in hypoxic VSMCs (Fig. 3D). We detected high levels of basal acetylation of histone H3 at K9/14 under normoxic conditions. Hypoxia remarkably reduced acetylation levels of histone H3 at K9/14, which were restored by Bur treatment (Fig. 3D). We then explored whether Bur induced the acetylation of histone H4 on the eNOS promoter region. We treated hypoxic VSMCs with 4 mM Bur and performed chromatin immunoprecipitation (ChIP) assays using an antibody specific for Ac‐H4‐K12/8/5/1. The results shown in Figure 3E indicate that a specific fragment of the eNOS promoter was amplified and detected in the precipitated Ac‐H4‐K12/8/5/1 complexes from the Bur‐treated hypoxic VSMCs. We also examined histone H3 acetylation on the eNOS promoter by the ChIP assay with an anti‐Ac‐histone H3‐K9/14 antibody, but no specific DNA fragment of the eNOS promoter was found in the immune complexes (data not shown). Taken together, these data further indicate that the HDI Bur activates eNOS gene expression in hypoxic VSMCs by epigenetically increasing the acetylation levels of histone H4 at its promoter region.

Taken together, our results indicate that the eNOS–NO system is activated by HDI in hypoxic VSMCs.

### eNOS expression in hypoxic VSMCs is required for HDI to suppress hypoxia‐induced cell growth

**Figure 4:**
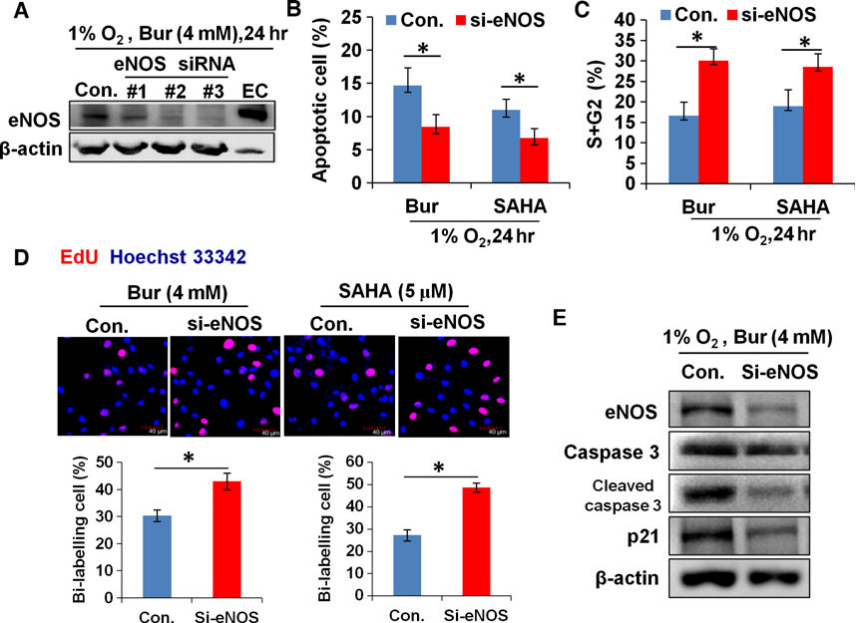
The antiproliferative and pro‐apoptotic effects of HDI on hypoxic VSMCs were weakened by eNOS siRNA transfection. (**A**) HDI‐induced expression of eNOS protein in hypoxic VSMCs was knocked down by eNOS siRNA transfection. β‐Actin protein was used as the sample loading control. Cell lysates from endothelial cell (EC) were used as the positive control. The experiment was performed in triplicate, and the representative data are shown. (**B**) The percentage of apoptotic VSMCs (indicated by positive annexin V staining and annexin V + PI double staining) was decreased remarkably in Bur‐ or SAHA‐treated hypoxic VSMCs upon eNOS siRNA transfection (*P < 0.05, *n* = 9). (**C**) Cell cycle analysis showed that the percentage of cells at the S and G2 phases was increased remarkably in both Bur‐ and SAHA‐treated hypoxic VSMCs upon eNOS siRNA transfection (*P < 0.05, *n* = 9). (**D**) The number of EdU^+^ cells was increased in both Bur‐ and SAHA‐treated hypoxic VSMCs upon eNOS siRNA transfection. Scale bar: 40 μm. The experiment was performed in triplicate, and the representative data are shown. (**E**) The protein levels of eNOS, caspase 3, cleaved caspase 3 and p21 were reduced in Bur‐treated hypoxic VSMCs that were transfected with eNOS siRNA. β‐Actin protein was used as the sample loading control.

To confirm the role of HDI‐induced eNOS expression on the proliferation and apoptosis of VSMCs, we used siRNA to specifically knock down eNOS in VSMCs. The efficiency of eNOS knockdown in hypoxic VSMCs is shown in Figure 4A, which indicated that all three siRNAs effectively knocked down eNOS expression at the protein level and that siRNA #3 had the best effect. Next, we used siRNA #3 to knock down eNOS expression in hypoxic VSMCs. Apoptosis assays showed that knockdown of eNOS decreased the percentage of VSMCs with annexin V staining and annexin V–PI double staining (Fig. 4B). Cell cycle analysis showed that knockdown of eNOS increased the percentage of VSMCs at the S and G2 phases (Fig. 4C). EdU incorporation assay also demonstrated that the number of EdU‐positive cells was increased in hypoxic VSMCs with eNOS siRNA transfection (Fig. 4D). Consistently, up‐regulation of cleaved caspase 3 and p21 by Bur was abrogated in hypoxic cells by eNOS siRNA transfection (Fig. 4E). These data demonstrate that induced eNOS expression in VSMCs is an essential biological process for Bur and SAHA to suppress hypoxia‐induced cell proliferation and to promote apoptosis.

### Induced secretion of NO by hypoxic VSMCs is required for HDI to suppress hypoxia‐induced cell growth

**Figure 5:**
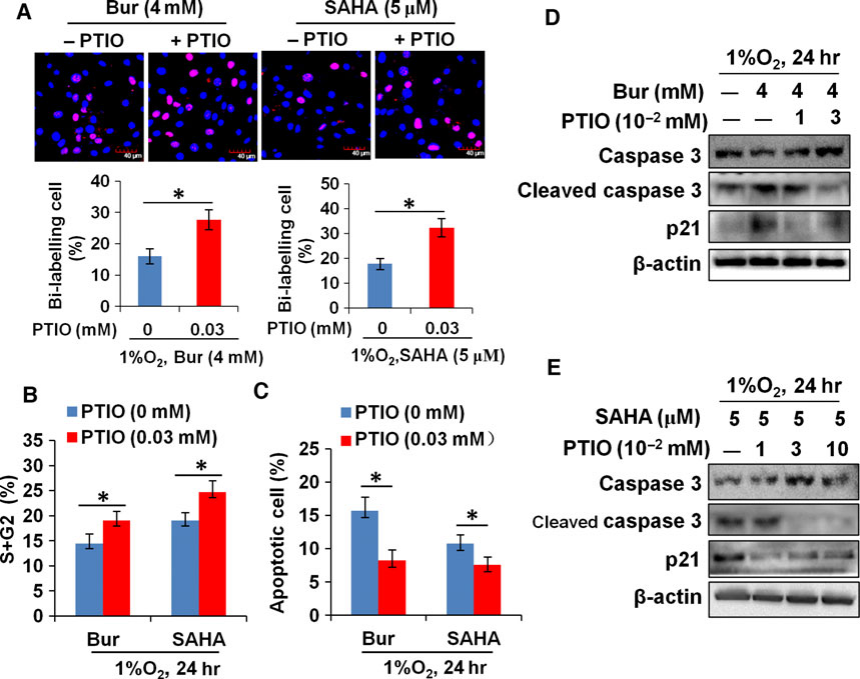
The NO scavenger carboxy‐PTIO restores the proliferation and survival of hypoxic VSMCs under HDI treatment. (**A**) The number of EdU^+^ cells was increased in both Bur‐ and SAHA‐treated hypoxic VSMCs upon the addition of the NO scavenger, carboxy‐PTIO. Scale bar: 40 μm. The experiment was performed in triplicate, and the representative image is shown. (**B**) Cell cycle analysis showed that the percentage of cells at the S and G2 phases was increased remarkably in both Bur‐ and SAHA‐treated hypoxic VSMCs upon the addition of the NO scavenger, carboxy‐PTIO (*P < 0.05, *n* = 5). (**C**) The percentage of apoptotic VSMCs (indicated by positive annexin V staining and annexin V + PI double staining) was decreased remarkably in Bur‐ or SAHA‐treated hypoxic VSMCs upon the addition of the NO scavenger, carboxy‐PTIO (*P < 0.05, *n* = 5). (**D**,** E**) HDI‐induced expression of cleaved caspase 3 and p21 was reduced in both Bur‐ and SAHA‐treated hypoxic VSMCs upon the addition of the NO scavenger, carboxy‐PTIO. β‐Actin protein was used as the sample loading control. The experiment was performed in triplicate, and the representative data are shown.

To explore whether NO secretion by hypoxic VSMCs is required for HDI to suppress hypoxia‐induced VSMC proliferation, we examined the effects of the NO scavenger carboxy‐PTIO on the antiproliferative and pro‐apoptotic properties of Bur and SAHA. As shown in Figure 5A and B, the addition of carboxy‐PTIO increased the number of EdU‐positive cells and the percentage of VSMCs at the S and G2 phases. However, the percentage of cells with annexin V staining and annexin V–PI double staining was decreased (Fig. 5C). Meanwhile, the level of cleaved caspase 3 and p21 was decreased in SAHA‐ or Bur‐treated hypoxic VSMCs upon carboxy‐PTIO addition (Fig. 5D). These data indicated that elevated NO secretion is required for Bur or SAHA to achieve its antiproliferative and pro‐apoptotic effects on hypoxic VSMCs.

### Bur attenuates pulmonary artery remodelling of rats exposed to chronic hypoxia

Hypoxia‐induced pulmonary arterial remodelling is characterized as thickening of medial layers of and collagen deposition at arterial walls. Both of them are related to VSMC activities 2, 3, 39. Hypoxia promotes proliferation and inhibits apoptosis of VSMCs, which contributes to excessive VSMC accumulation in the artery wall and to the thickening. Nevertheless, hypoxia induces a phenotype transformation of VSMCs from a contractile, differentiated to a synthetic, dedifferentiated state, which contributes to increased proliferative ability and collagen oversynthesis of VSMCs. To determine the effect of Bur on hypoxia‐induced VSMC accumulation *in vivo*, we established a rat model of pulmonary artery remodelling by exposing the rats to hypobaric hypoxia for 28 days. The thickness and collagen deposition of pulmonary arteries were then estimated. The greatest influence of pulmonary arterial wall thickness is the increase in pulmonary vascular resistance and right ventricular afterload, reflected by the elevation of mean pulmonary arterial pressure (mPAP) and the right ventricle hypertrophy index (RVHI, the ratio of right ventricle weight to left ventricle plus septum weight). To assess the function of pulmonary arteries, mPAP, RVHI and PaO_2_ were evaluated.

**Figure 6:**
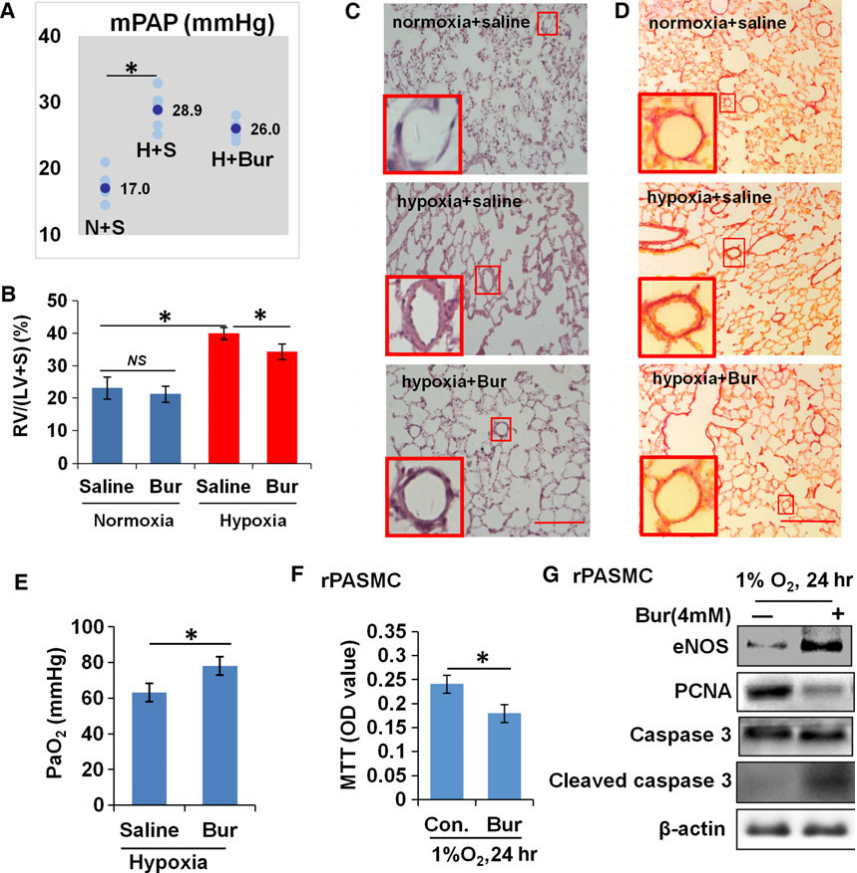
Bur abrogates hypoxia‐induced pulmonary artery remodelling. (**A**) Pulmonary arterial pressure was significantly elevated in rats under long‐term exposure (28 days) to hypobaric hypoxia (*n* = 6), but showed no obvious changes in hypoxic rats with Bur administration (*n* = 6). N + S, H + S and H + Bur indicate normoxia + saline, hypoxia + saline and hypoxia + Bur groups, respectively. The average value of mPAP in each group is shown. (**B**) Right ventricle hypertrophy index (RVHI) was significantly decreased in hypoxic rats upon Bur administration (*n* = 6). (**C**) H&E staining analysis showed that long‐term exposure (28 days) to hypobaric hypoxia increased the thickness of the pulmonary arterial wall in rats, which was partly prevented by Bur administration. Scale bar: 100 μm. Each experiment was performed in three cases, and the representative data are shown. (**D**) Sirius Red staining showing that exposure to hypobaric hypoxia promoted collagen deposition at the pulmonary arterial wall rats, which was prevented upon Bur administration. Scale bar: 100 μm. Each experiment was performed in three cases, and the representative is shown. (**E**) Bur administration protected the lung function in rats exposed to long‐term hypobaric hypoxia as indicated by the increase in PaO_2_ (*P < 0.05, *n* = 7). (**F**) Cell viability of hypoxic PASMCs was decreased upon Bur (4 mM) treatment (*P < 0.05, *n* = 9). (**G**) The protein level of PCNA was reduced in hypoxic PASMCs upon the addition of Bur (4 mM), while the levels of eNOS and cleaved caspase 3 were increased. β‐Actin protein was used as the sample loading control. The experiment was performed in triplicate, and the representative image is shown.

As shown in Figure 6A, the mPAP of hypoxic rats was much higher than that in normoxic rats. Consistently, the RVHI was increased with exposure to hypoxia (Fig. 6B). Similarly, rats exposed to hypobaric hypoxia showed increased thickness in the pulmonary artery wall when compared with that rats under normal conditions (308 m above sea level, Chongqing, China; Fig. 6C). Rats exposed to hypobaric hypoxia showed obvious collagen deposition at the pulmonary artery, which was barely observed in the control rat group (Fig. 6D). However, the thickness and collagen deposition of the pulmonary artery wall were less obvious in Bur‐treated hypoxic rats (Fig. 6C and D). Although mPAP showed no obvious changes in hypoxic rats with Bur administration, the RVHI was decreased significantly (Fig. 6A and B). Simultaneously, PaO_2_ was increased in rats exposed to long‐term hypobaric hypoxia and administrated Bur (Fig. 6E). To evaluate the effect of Bur on the viability of pulmonary arterial SMCs (PASMCs), we treated primarily cultured PASMCs with 4 mM Bur and cultured them in a hypoxic incubator for 24 hr. MTT assay showed that Bur treatment significantly reduced the viability of PASMCs (Fig. 6F). The protein levels of eNOS and cleaved caspase 3 in Bur‐treated hypoxic PASMCs were elevated (Fig. 6G). These results further suggest that Bur may effectively suppress hypoxia‐induced vascular remodelling *in vivo*.

## Discussion

HDIs have been widely used as an antiproliferation reagent in cancer research. Some of these such as Bur and SAHA have been tested in clinical trials. However, the effect of HDI on hypoxia‐induced VSMC proliferation is still unknown. In this study, we demonstrate that Bur and SAHA suppress the proliferation and promote apoptosis in hypoxic VSMCs *in vitro*. *In vivo*, the administration of Bur in rats exposed to hypobaric hypoxia for 28 days ameliorated the thickness and collagen deposition of pulmonary artery walls. These results indicate that Bur and SAHA are effective for suppressing hypoxia‐induced VSMC growth and prevent the development of vascular remodelling.

Previous studies reported that trichostatin A, another HDI, also suppresses VSMC proliferation 14, 40, 41. However, its definite cytotoxicity restricts its application potential 14, 40, 42. Alternatively, Bur is a naturally occurring short‐chain fatty acid and physiologically exists in the intestinal system and blood. It is a major metabolite of fibre‐enriched food 43, 44, 45, 46, 47, 48. Recent studies demonstrated the safety of butyrate in the protection of the cardiovascular system 32. SAHA is another HDI that is well tolerated intravenously 49. It has been approved by the FDA as an anticancer drug 50. In our study, we confirmed that Bur and SAHA are safe because they have no influence on the basal growth of normoxic VSMCs at low concentrations. Bur and SAHA are primarily proven to be anti‐inflammatory reagents and are effective at preventing the development of inflammatory bowel disease and lung fibrosis 8, 48, 50, 51, 52, 53. Our data demonstrate a novel application for these HDIs in maintaining cardiovascular homoeostasis.

The main clinical signs of PH include increased right ventricular systolic pressure, increased right ventricular weight and considerable pulmonary vascular remodelling 54. In this study, we found that vascular remodelling and right ventricular hypertrophy were partly suppressed by Bur administration in hypoxic rats. Unfortunately, a significant decrease in mPAP was not observed, although the average value appeared to have a declining trend in hypoxic rats upon Bur administration, which is unexpected but reasonable. The level of mPAP is dependent not only on the function of the pulmonary artery system but also on right ventricular function. Although elevated mPAP is an important contributor to right ventricular hypertrophy, direct responses of heart cells to hypoxia are also essential for cardiac remodelling. It has been revealed that hypoxia‐induced pulmonary vascular remodelling of rats was reversed after they were freed from the hypoxic environment for up to 6 weeks. However, significant recovery of PAP, right ventricular systolic pressure and right ventricular hypertrophy was not observed simultaneously 54, 55. In this regard, although HDIs show no obvious inhibition of hypoxia‐induced mPAP elevation, we believe that HPH development is suppressed by HDIs, which is reflected by the reversal of histological changes in the cardiovascular system. Further analysis of the benefit and feasibility of HDIs in the prevention of hypoxia‐induced vascular disorders needs to be properly defined in the future.

Hypoxia‐induced VSMC proliferation is associated with endogenous activity of VSMCs and the factors secreted by neighbouring cells or those transported from blood 30, 56, 57. Although growth factors, including PDGF, are contributors to hypoxia‐induced VSMC proliferation 58, 59, 60, 61, 62, they are mainly derived from either vascular endothelial cells or migrated inflammatory cells 56, 61, 63. Although HDIs suppressed PDGF‐induced VSMC proliferation, autosecretion of PDGF was not detected when VSMC was stimulated by hypoxia alone 63, 64. In this study, cultured VSMCs were free from the effects of other cell types or systemic responses to hypoxia. Nevertheless, we found that Bur and SAHA had no influence on the baseline of VSMC proliferation under normoxia at the concentration used in this study. Our data suggest for the first time that HDIs may inhibit hypoxia‐induced VSMC growth through a mechanism that is endogenous to VSMCs and independent of external sources of factors, such as PDGF.

As described in other reports, three functional classes of genes were dominantly changed by HDIs in VSMCs, which were associated with cell growth and differentiation, stress response or vascular function 10, 11. Examination of differentially expressed cell growth‐ and differentiation‐related genes indicated that butyrate‐inhibited VSMC proliferation appears to involve down‐regulation of genes that encode several positive regulators of cell growth and up‐regulation of some negative regulators of growth or differentiation inducers 10. Analysis of histone H3 modifications specific to butyrate‐arrested VSMC proliferation displayed induction of histone H3‐lysine 9 acetylation, inhibition of histone H3‐serine 10 phosphorylation, reduction of histone H3‐lysine 9 dimethylation and stimulation of histone H3‐lysine 4 dimethylation 13. The interplay of these site modifications might cause distinct chromatin alterations that allow distinct gene expression patterns 13. However, the epigenetic mechanism of HDI on cell growth regulation is still largely unknown.

In this study, we proved that endogenous eNOS expression was induced by Bur and SAHA treatment in hypoxic VSMCs. It has been reported that hyperacetylation of histone H4‐lysine 12 and H3‐lysine 9 is specifically related to eNOS expression in vascular endothelial cells. Increased acetylation levels at these sites are positively correlated with activation of the eNOS gene in non‐endothelial cells, such as cardiac cells 15, 16. We noticed that Bur treatment increased histone acetylation on the eNOS promoter in hypoxic VSMCs. However, only the hyperacetylation of histone H4‐lysine 12/8/5/1 was detected on the eNOS promoter in Bur‐treated hypoxic VSMCs, while acetylation of histone H3 at lysine 9 was undetected. Reduced global histone H4 acetylation and DNA methylation in the pulmonary vasculature of foetal lambs exposed to high‐altitude (3180 m) hypoxia was accompanied by the loss of p21 expression and contributed to SMC proliferation. These results suggest that acetylation of histone H4 at lysine 12/8/5/1 was remarkably induced by Bur in hypoxic VSMCs and was related to eNOS activation. It also implies that like other HDIs, Bur and SAHA may have biological effects on VSMC growth along with other mechanisms as well. Being the first such study, the role of HDI‐induced eNOS expression on cell growth was further analysed to facilitate a better understanding of the mechanism underlying HDI‐mediated protection of the cardiovascular system. Although the acetylation of histone H3 at lysine 9 was alleviated dramatically under hypoxia but was restored by Bur as declared by other reports, it did not participate in eNOS activation. However, it is widely believed that hypoacetylation of histone H3 at lysine 9 is closely related to gene repression. Primary studies had shown that p21 expression was suppressed by hypoxia and was restored by HDIs 10, 13, 14, 30, 42. Interestingly, we found that the alteration of p21 expression was positively correlated with the variation of histone H3 acetylation at lysine 9. Therefore, future studies on the modification of histone H3 at lysine 9 are necessary for a better understanding of the mechanism mediating hypoxia‐induced suppression of p21.

In physiological conditions, eNOS‐derived NO is secreted by vascular endothelial cells and this migrates into VSMCs. The effect of NO on VSMC growth is based on two main mechanisms. The first is to promote the cleavage of caspase 3, which contributes to apoptosis 65. The other mechanism is to promote p21 expression, which inhibits cell cycle progression 28, 65. We found that decreased proliferation and increased apoptosis induced by HDI treatment were alleviated by an NO scavenger or upon inhibition of eNOS expression in hypoxic VSMCs. Consistently, HDI‐induced expression of cleaved caspase 3 and p21 was also attenuated. These data suggest that induction of exogenous eNOS–NO activity in hypoxic VSMCs was directly involved in the antiproliferative and pro‐apoptotic effects of HDI. It has been mentioned above that HDI‐inhibited VSMC proliferation was based on the combined action of genes that serve as positive or negative regulators of cell growth 10, 11. Therefore, the positive integrators that combine the signals from all regulators are essential for understanding the protective role of HDI. A future study would be needed to investigate whether induced eNOS expression and bioactive NO secretion in VSMCs act as the combined mediator for HDI in cell growth regulation.

In many pathological conditions, eNOS‐derived NO production is attenuated 20, 24, 25. Due to endothelial cell injury and eNOS dysfunction, restoration of the endothelium‐based eNOS–NO system in pathological circumstances is limited and difficult 20, 23, 66. It has been demonstrated that eNOS transfection in adipose‐derived stem cells results in bioactive nitric oxide production 67. Another report has declared that eNOS gene transfection in VSMCs inhibits cell proliferation *via* up‐regulation of p27 and p21, resulting in delayed cell cycle progression, but no effect on apoptosis 26, 68, 69. Our study also proved that endogenous expression of eNOS in hypoxic VSMCs is achievable and effective for cell cycle suppression and apoptosis promotion. Interestingly, our study provides data to suggest an epigenetic strategy that activates endogenous eNOS–NO system in hypoxic VSMCs that can be easily realized by treatment with Bur and SAHA, two HDIs that have been approved clinically or are currently under clinical trials.

**Figure 7:**
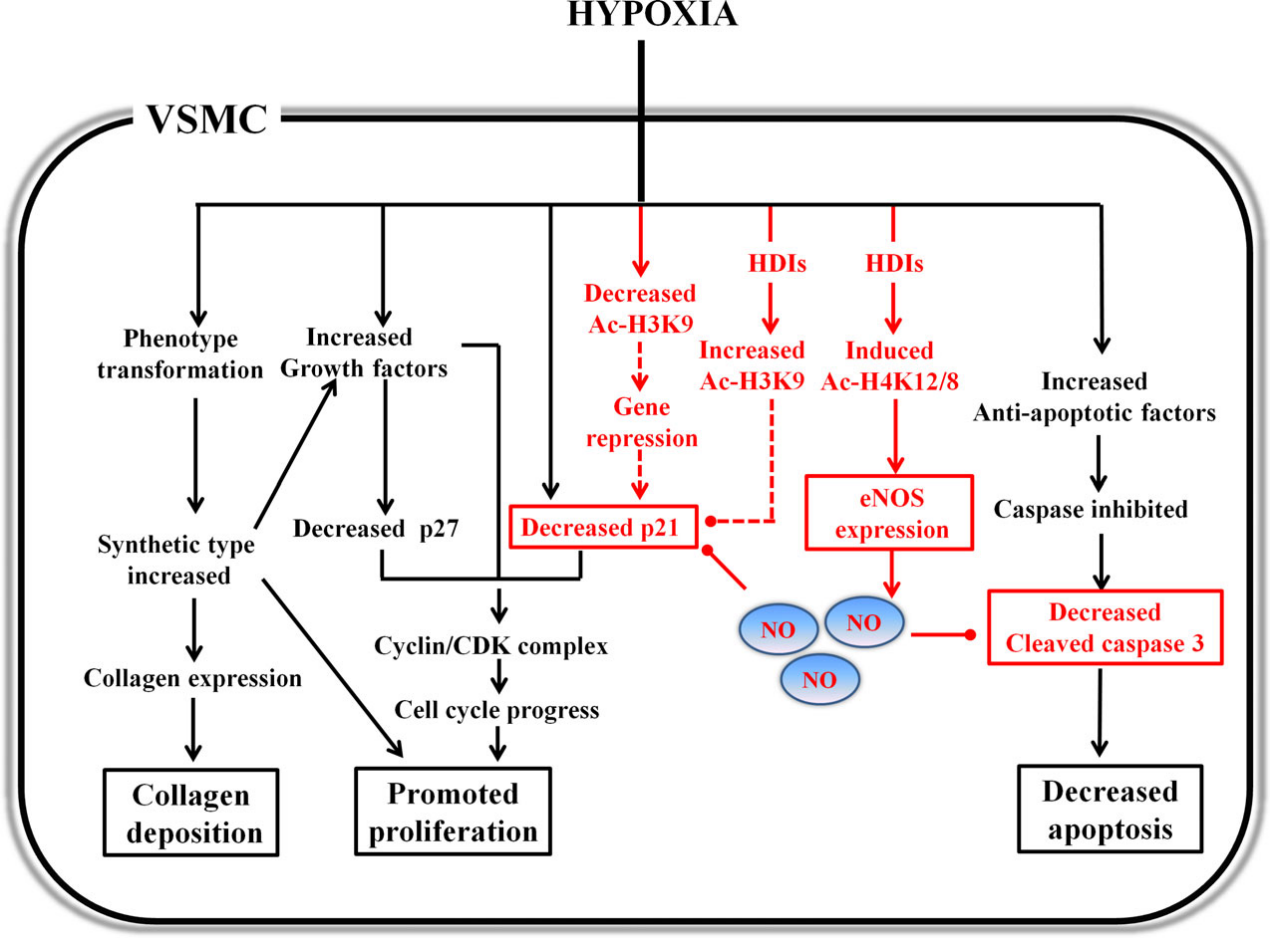
A schematic summary of the suppression of hypoxia‐induced VSMC growth by HDIs involving eNOS–NO activation. The point arrows are used to indicate promotion effects. The round‐headed arrows are used to indicate suppression. The parts with dotted lines are shown as speculative views. The abbreviations are described as follows: HDI, histone deacetylase inhibitor; Ac‐H3K9, acetylated histone H3 at lysine 9; Ac‐H4K12/8, acetylated histone H4 at lysine 12/lysine 8; eNOS, endothelial nitric oxide synthase.

As schematically summarized in Figure 7, the HDIs, Bur and SAHA, activate the eNOS–NO system in hypoxic VSMCs. Increased NO production may mediate HDI‐induced cell cycle arrest and apoptosis. Our findings not only indicate a novel mechanism by which HDI suppresses hypoxia‐triggered VSMC growth, but also pave a possibility for the clinical utilization of HDIs, Bur and SAHA, to prevent vascular remodelling related to hypoxic stimulation.

## Conflict of interest

The authors confirm that there are no conflict of interests.

## Funding

The work was supported by the National Natural Science Foundation of China (grant numbers: 81270108; 30971198) and the National Basic Research Program of China (973 Program, grant number: 2012CB518201).
